# Crystal structure of 11-[4-(hex­yloxy)phen­yl]-1,2,4-triazolo[4,3-*a*][1,10]phenanthroline

**DOI:** 10.1107/S2056989015012025

**Published:** 2015-06-27

**Authors:** Jasmin Preis, Dieter Schollmeyer, Heiner Detert

**Affiliations:** a University Mainz, Duesbergweg 10-14, 55099 Mainz, Germany

**Keywords:** crystal structure, phenanthroline, triazole, helicene

## Abstract

The title compound, C_25_H_24_N_4_O, was prepared from 2-chloro­phenanthroline and hexyl­oxyphenyl­tetra­zole. The main difference between the two independent mol­ecules (*A* and *B*) in the asymmetric unit is the orientation of the all-*anti-*configured hex­yloxy chain: in *A* the C—O—C—C torsion angle is 175.9 (2)° whereas it is −88.3 (3)° in *B*. The benzene substitution in the bay of the triazolophenanthroline results in a helical distorsion of the heterocyclic core, the dihedral angles between the mean planes formed by quinoline and benzotriazole ring systems are 13.73 (9) for mol­ecule *A* and 14.87 (8)° for B. The dihedral angles between the triazole ring and the attached benzene ring are 45.87 (15) in *A* and 53.93 (14)° in *B*. The angular annulation of four rings and the benzene substituent results in a helical distortion of the aromatic framework. The crystal is formed from layers composed of centrosymmetric pairs of *A*_2_, *B*_2_ mol­ecules with inter­digitating alkyl chains.

## Related literature   

For structures of 1,2,4-triazolo annulated diazines, see: Preis *et al.* (2011*a* ▸,*b* ▸); for a triazolo­thia­zole, see: Schollmeyer & Detert (2014 ▸); for threefold triazoloannulated triazines, see: Cristiano *et al.* (2008 ▸); Herget *et al.* (2013 ▸); Glang *et al.* (2014 ▸); Rieth *et al.* (2014 ▸). For structures of aza­helicenes, see: Caronna *et al.* (2012 ▸); Upadhyay *et al.* (2014 ▸). Synthesis: for chloro­phenanthroline as starting material, see: Lewis & O’Donoghue (1980 ▸); for 1,2,4-triazoloannulation *via* tetra­zoles, see: Huisgen *et al.* (1960 ▸).
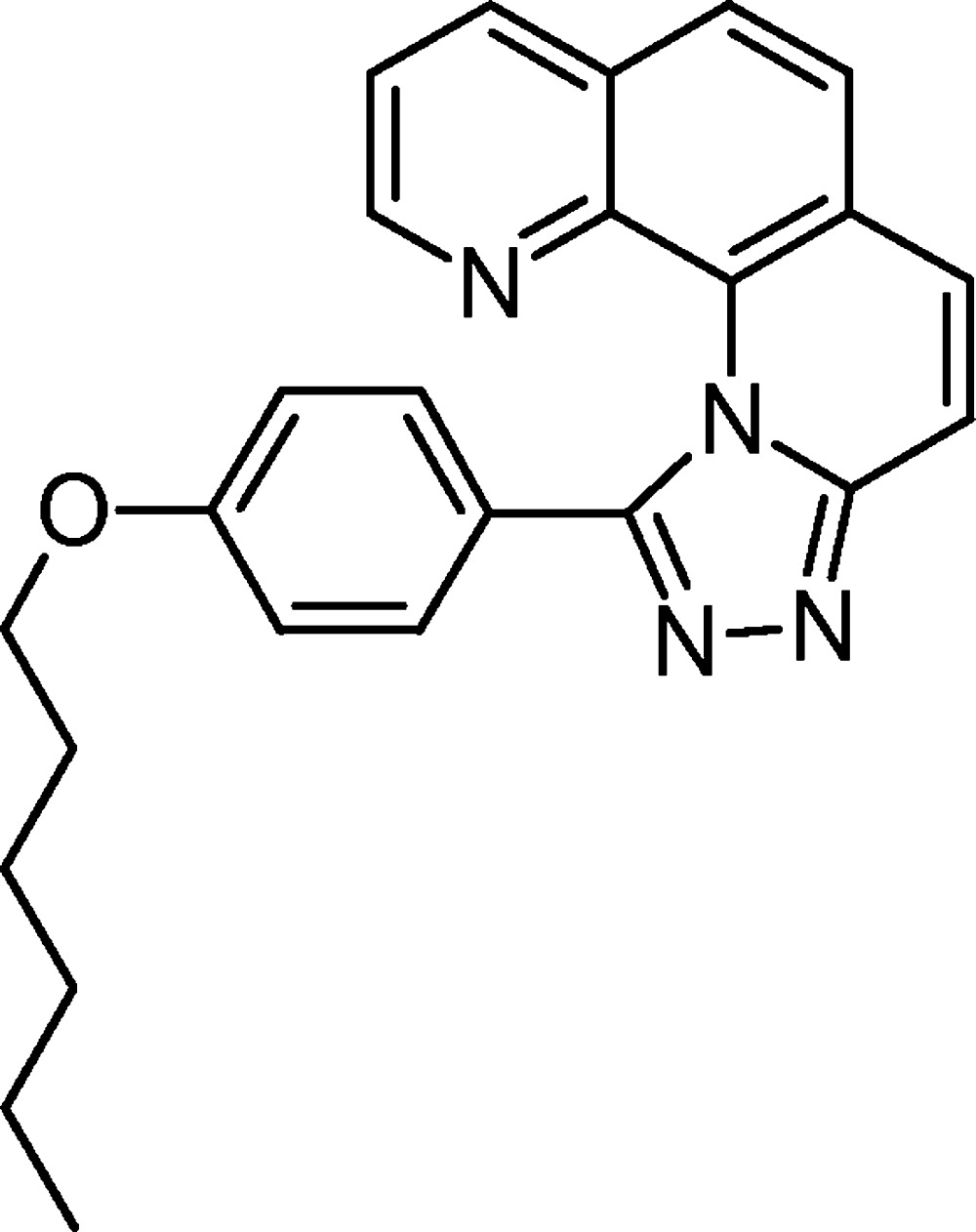


## Experimental   

### Crystal data  

C_25_H_24_N_4_O
*M* *_r_* = 396.48
Monoclinic, 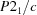

*a* = 9.746 (3) Å
*b* = 36.787 (5) Å
*c* = 12.174 (3) Å
β = 106.412 (12)°
*V* = 4186.6 (17) Å^3^
*Z* = 8
Cu *K*α radiation
μ = 0.62 mm^−1^
*T* = 193 K
0.34 × 0.23 × 0.23 mm

### Data collection  

Enraf–Nonius CAD-4 diffractometer
8415 measured reflections
7934 independent reflections
5140 reflections with *I* > 2σ(*I*)
*R*_int_ = 0.051
3 standard reflections every 60 min intensity decay: 5%

### Refinement  

*R*[*F* ^2^ > 2σ(*F* ^2^)] = 0.063
*wR*(*F* ^2^) = 0.170
*S* = 1.02
7934 reflections
543 parameters
H-atom parameters constrained
Δρ_max_ = 0.41 e Å^−3^
Δρ_min_ = −0.31 e Å^−3^

### 

Data collection: *CAD-4 Software* (Enraf–Nonius, 1989 ▸); cell refinement: *CAD-4 Software*; data reduction: *CORINC* (Dräger & Gattow, 1971 ▸); program(s) used to solve structure: *SIR97* (Altomare *et al.*, 1999 ▸); program(s) used to refine structure: *SHELXL2014* (Sheldrick, 2015 ▸); molecular graphics: *PLATON* (Spek, 2009 ▸); software used to prepare material for publication: *SHELXL2014*.

## Supplementary Material

Crystal structure: contains datablock(s) I, New_Global_Publ_Block. DOI: 10.1107/S2056989015012025/nr2059sup1.cif

Structure factors: contains datablock(s) I. DOI: 10.1107/S2056989015012025/nr2059Isup2.hkl

Supporting information file. DOI: 10.1107/S2056989015012025/nr2059Isup3.cml

**Fig. 1.**
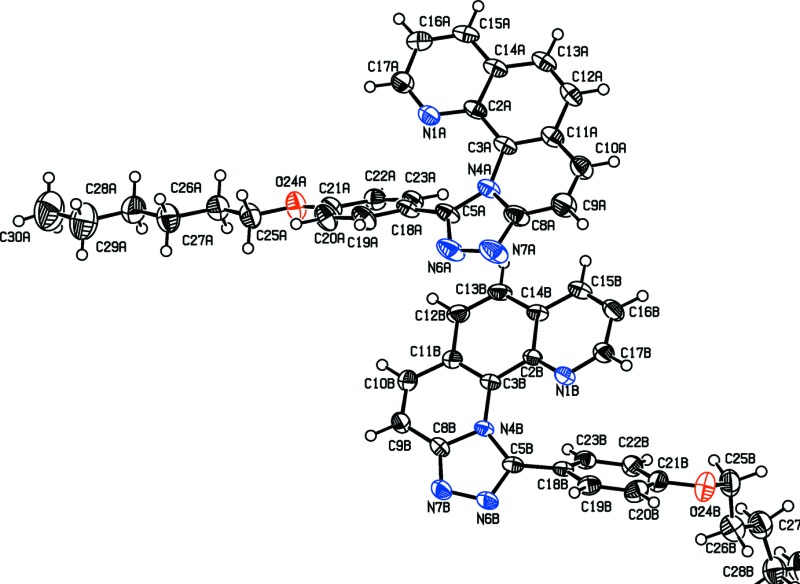
Crystal structure of the title compound with labeling and displacement ellipsoids drawn at the 50% probability level.

. DOI: 10.1107/S2056989015012025/nr2059fig1.tif
Crystal structure of the title compound with labeling and displacement ellipsoids drawn at the 50% probability level.

**Fig. 2.**
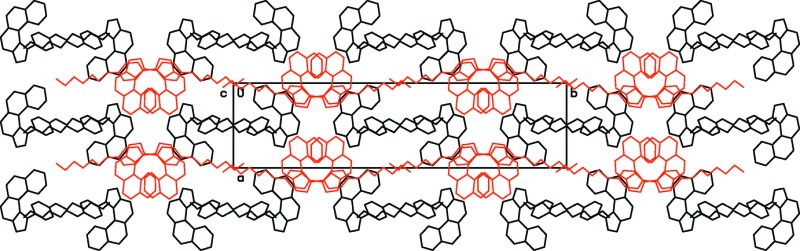
Part of the packing diagram. Molecule B coloured in red. View along the c axis.

B c . DOI: 10.1107/S2056989015012025/nr2059fig2.tif
Part of the packing diagram. Mol­ecule *B* coloured in red. View along the *c* axis.

CCDC reference: 1408324

Additional supporting information:  crystallographic information; 3D view; checkCIF report

## supplementary crystallographic information

### S1. Chemical context

The title compound, C_25_H_24_N_4_O, was prepared as part of a larger study on triazolo-annulated azines, see Glang *et al.* (2014), Rieth *et al.* (2014), Preis *et al.* (2011a). This triazolophenanthroline was prepared according to Huisgen (Huisgen *et al.* 1960) via nucleophilic substitution, cyclo­elimination/ring closure from 2-chloro­phenanthroline (Lewis & O'Donoghue, 1980) and hexyl­oxyphenyl tetra­zole. This is an efficient method for the preparation of triazolo-annulated azines.

### S2. Structural commentary

The monoclinic unit cell is composed of two different molecules A, B of the title compound.The main difference between A and B is the orientation of the *all-anti* configured hexyl­oxy chain: in A the torsion angle C21A—O24A—C25A—C26A is 175.9 (2)° wheras C21B—O24B—C25B—C26B is -88.3 (3)°. The phenyl substitution in the bay of the triazolophenanthroline results in a helical distorsion of the heterocyclic core, the dihedral angles between the mean planes formed by quinoline and benzotriazole are 13.73 (9) ° for molecule A and 14.87 (8) ° for B. The dihedral angles between the triazole ring and the attached phenyl ring are 45.87 (15) ° (A) and 53.93 (14) ° (B).

### S3. Supra­molecular features

The crystal is formed from layers composed of centrosymmetric pairs of A_2_, B_2_ and the alkyl chains are inter­digitating.

### S4. Synthesis and crystallization

The title compound was prepared by refluxing a solution of 5-(4-hexyl­oxyphenyl)­tetra­zole (150 mg, 0.61 mmol) and 2-chloro-[1,10]phenanthroline ( 131 mg, 0.61 mmol), prepared according to Lewis & O'Donoghue (Lewis & O'Donoghue, 1980) in xylenes / pyridine (7mL, 5/1) for 8 d. The solvents were evaporated and the residue purified via chromatography on silica gel using ethyl acetate / toluene (2/1) with 1% tri­ethyl­amine as an eluent. Yield: 128 mg (53%) of a brownish solid with m. p.= 419 K. Single crystals were obtained by slow evaporation of a saturated solution in chloro­form/ethanol (5/1).

### S5. Refinement details

Crystal data, data collection and structure refinement details are summarized in Table 1. Hydrogen atoms attached to carbons were placed at calculated positions with C—H = 0.95 Å (aromatic) or 0.98–0.99 Å (*sp*^3^ C-atom). All H atoms were refined in the riding-model approximation with isotropic displacement parameters (set at 1.2–1.5 times of the *U*_eq_ of the parent atom).

### Crystal data

**Table:**

C_25_H_24_N_4_O	*F*(000) = 1680
*M**_r_* = 396.48	*D*_x_ = 1.258 Mg m^−^^3^
Monoclinic, *P*2_1_/*c*	Cu *K*α radiation, λ = 1.54178 Å
*a* = 9.746 (3) Å	Cell parameters from 25 reflections
*b* = 36.787 (5) Å	θ = 25–38°
*c* = 12.174 (3) Å	µ = 0.62 mm^−^^1^
β = 106.412 (12)°	*T* = 193 K
*V* = 4186.6 (17) Å^3^	Block, colourless
*Z* = 8	0.34 × 0.23 × 0.23 mm

### Data collection

**Table:**

Enraf–Nonius CAD-4 diffractometer	*R*_int_ = 0.051
Radiation source: rotating anode	θ_max_ = 70.2°, θ_min_ = 2.4°
Graphite monochromator	*h* = −11→0
ω/2θ scans	*k* = 0→44
8415 measured reflections	*l* = −14→14
7934 independent reflections	3 standard reflections every 60 min
5140 reflections with *I* > 2σ(*I*)	intensity decay: 5%

### Refinement

**Table:**

Refinement on *F*^2^	0 restraints
Least-squares matrix: full	Hydrogen site location: inferred from neighbouring sites
*R*[*F*^2^ > 2σ(*F*^2^)] = 0.063	H-atom parameters constrained
*wR*(*F*^2^) = 0.170	*w* = 1/[σ^2^(*F*_o_^2^) + (0.0793*P*)^2^ + 0.8479*P*] where *P* = (*F*_o_^2^ + 2*F*_c_^2^)/3
*S* = 1.02	(Δ/σ)_max_ < 0.001
7934 reflections	Δρ_max_ = 0.41 e Å^−^^3^
543 parameters	Δρ_min_ = −0.30 e Å^−^^3^

### Special details

**Table:**

Geometry. All e.s.d.'s (except the e.s.d. in the dihedral angle between two l.s. planes) are estimated using the full covariance matrix. The cell e.s.d.'s are taken into account individually in the estimation of e.s.d.'s in distances, angles and torsion angles; correlations between e.s.d.'s in cell parameters are only used when they are defined by crystal symmetry. An approximate (isotropic) treatment of cell e.s.d.'s is used for estimating e.s.d.'s involving l.s. planes.

### Fractional atomic coordinates and isotropic or equivalent isotropic displacement parameters (Å^2^)

**Table:**

	*x*	*y*	*z*	*U*_iso_*/*U*_eq_	
N1A	0.3086 (2)	0.08512 (6)	0.6267 (2)	0.0381 (5)	
C2A	0.2720 (3)	0.11722 (8)	0.6648 (2)	0.0331 (6)	
C3A	0.3790 (3)	0.14428 (8)	0.7072 (2)	0.0334 (6)	
N4A	0.5224 (2)	0.14042 (6)	0.70355 (18)	0.0358 (5)	
C5A	0.5963 (3)	0.11790 (8)	0.6480 (2)	0.0391 (7)	
N6A	0.7342 (3)	0.12405 (8)	0.6896 (2)	0.0551 (7)	
N7A	0.7563 (3)	0.15103 (9)	0.7702 (2)	0.0612 (8)	
C8A	0.6297 (3)	0.16150 (9)	0.7758 (2)	0.0462 (7)	
C9A	0.5964 (3)	0.19102 (9)	0.8380 (3)	0.0528 (8)	
H9A	0.6702	0.2054	0.8860	0.063*	
C10A	0.4585 (3)	0.19833 (9)	0.8279 (3)	0.0461 (7)	
H10A	0.4340	0.2194	0.8636	0.055*	
C11A	0.3481 (3)	0.17474 (8)	0.7640 (2)	0.0392 (6)	
C12A	0.2039 (3)	0.18070 (8)	0.7658 (2)	0.0431 (7)	
H12A	0.1815	0.2021	0.8008	0.052*	
C13A	0.0992 (3)	0.15688 (8)	0.7195 (2)	0.0416 (7)	
H13A	0.0039	0.1619	0.7205	0.050*	
C14A	0.1303 (3)	0.12420 (8)	0.6692 (2)	0.0359 (6)	
C15A	0.0257 (3)	0.09793 (8)	0.6239 (2)	0.0417 (7)	
H15A	−0.0706	0.1020	0.6236	0.050*	
C16A	0.0623 (3)	0.06658 (8)	0.5803 (3)	0.0441 (7)	
H16A	−0.0079	0.0488	0.5474	0.053*	
C17A	0.2064 (3)	0.06120 (8)	0.5851 (3)	0.0449 (7)	
H17A	0.2320	0.0389	0.5567	0.054*	
C18A	0.5455 (3)	0.09471 (8)	0.5466 (2)	0.0354 (6)	
C19A	0.6156 (3)	0.06239 (8)	0.5409 (3)	0.0447 (7)	
H19A	0.6905	0.0547	0.6052	0.054*	
C20A	0.5791 (3)	0.04110 (9)	0.4442 (3)	0.0485 (8)	
H20A	0.6284	0.0189	0.4425	0.058*	
C21A	0.4708 (3)	0.05184 (8)	0.3494 (2)	0.0402 (7)	
C22A	0.4013 (3)	0.08460 (7)	0.3532 (2)	0.0373 (6)	
H22A	0.3276	0.0924	0.2883	0.045*	
C23A	0.4382 (3)	0.10573 (8)	0.4497 (2)	0.0366 (6)	
H23A	0.3903	0.1282	0.4508	0.044*	
O24A	0.4242 (2)	0.03266 (6)	0.24946 (18)	0.0507 (6)	
C25A	0.4945 (3)	−0.00070 (8)	0.2399 (3)	0.0474 (7)	
H25A	0.4920	−0.0172	0.3036	0.057*	
H25B	0.5957	0.0039	0.2433	0.057*	
C26A	0.4169 (3)	−0.01768 (8)	0.1266 (3)	0.0483 (7)	
H26A	0.4176	−0.0006	0.0640	0.058*	
H26B	0.3160	−0.0222	0.1244	0.058*	
C27A	0.4855 (4)	−0.05310 (9)	0.1076 (3)	0.0560 (9)	
H27A	0.5856	−0.0481	0.1081	0.067*	
H27B	0.4885	−0.0695	0.1727	0.067*	
C28A	0.4121 (4)	−0.07244 (10)	−0.0013 (3)	0.0618 (9)	
H28A	0.4075	−0.0560	−0.0667	0.074*	
H28B	0.3127	−0.0780	−0.0014	0.074*	
C29A	0.4867 (5)	−0.10793 (12)	−0.0186 (4)	0.0866 (13)	
H29A	0.5842	−0.1021	−0.0226	0.104*	
H29B	0.4963	−0.1237	0.0492	0.104*	
C30A	0.4130 (6)	−0.12808 (13)	−0.1203 (4)	0.1080 (18)	
H30A	0.3191	−0.1358	−0.1143	0.162*	
H30B	0.4695	−0.1495	−0.1275	0.162*	
H30C	0.4004	−0.1126	−0.1878	0.162*	
N1B	0.9406 (2)	0.24530 (6)	0.63224 (18)	0.0328 (5)	
C2B	0.8565 (2)	0.22341 (7)	0.5515 (2)	0.0274 (5)	
C3B	0.9214 (2)	0.20124 (7)	0.4832 (2)	0.0286 (5)	
N4B	1.0647 (2)	0.20604 (6)	0.48078 (17)	0.0278 (4)	
C5B	1.1665 (3)	0.23334 (7)	0.5133 (2)	0.0309 (5)	
N6B	1.2883 (2)	0.22181 (6)	0.4984 (2)	0.0377 (5)	
N7B	1.2699 (2)	0.18737 (6)	0.4529 (2)	0.0379 (5)	
C8B	1.1352 (3)	0.17842 (7)	0.4402 (2)	0.0314 (6)	
C9B	1.0598 (3)	0.14781 (7)	0.3835 (2)	0.0359 (6)	
H9B	1.1079	0.1292	0.3549	0.043*	
C10B	0.9179 (3)	0.14563 (7)	0.3709 (2)	0.0367 (6)	
H10B	0.8644	0.1262	0.3279	0.044*	
C11B	0.8468 (3)	0.17217 (7)	0.4214 (2)	0.0321 (6)	
C12B	0.6978 (3)	0.16838 (8)	0.4115 (2)	0.0395 (6)	
H12B	0.6455	0.1489	0.3680	0.047*	
C13B	0.6296 (3)	0.19222 (8)	0.4634 (2)	0.0393 (6)	
H13B	0.5291	0.1904	0.4509	0.047*	
C14B	0.7071 (3)	0.21971 (7)	0.5358 (2)	0.0329 (6)	
C15B	0.6446 (3)	0.24288 (8)	0.5998 (2)	0.0413 (7)	
H15B	0.5444	0.2421	0.5899	0.050*	
C16B	0.7283 (3)	0.26637 (8)	0.6758 (3)	0.0443 (7)	
H16B	0.6871	0.2828	0.7176	0.053*	
C17B	0.8769 (3)	0.26598 (8)	0.6916 (2)	0.0389 (6)	
H17B	0.9348	0.2815	0.7484	0.047*	
C18B	1.1488 (2)	0.27117 (7)	0.5436 (2)	0.0296 (5)	
C19B	1.2452 (3)	0.28641 (8)	0.6394 (2)	0.0377 (6)	
H19B	1.3193	0.2719	0.6866	0.045*	
C20B	1.2332 (3)	0.32247 (8)	0.6658 (2)	0.0429 (7)	
H20B	1.2988	0.3327	0.7317	0.052*	
C21B	1.1261 (3)	0.34398 (7)	0.5969 (2)	0.0387 (6)	
C22B	1.0320 (3)	0.32928 (7)	0.4996 (2)	0.0355 (6)	
H22B	0.9598	0.3440	0.4509	0.043*	
C23B	1.0443 (3)	0.29311 (7)	0.4743 (2)	0.0326 (6)	
H23B	0.9796	0.2830	0.4078	0.039*	
O24B	1.1212 (3)	0.37890 (6)	0.63403 (19)	0.0546 (6)	
C25B	1.0265 (4)	0.40429 (8)	0.5614 (3)	0.0552 (9)	
H25C	0.9961	0.4226	0.6092	0.066*	
H25D	0.9400	0.3913	0.5160	0.066*	
C26B	1.0954 (4)	0.42309 (10)	0.4818 (3)	0.0566 (9)	
H26C	1.1232	0.4048	0.4325	0.068*	
H26D	1.1836	0.4354	0.5272	0.068*	
C27B	0.9968 (4)	0.45097 (9)	0.4066 (3)	0.0592 (9)	
H27C	0.9221	0.4380	0.3475	0.071*	
H27D	0.9483	0.4649	0.4543	0.071*	
C28B	1.0725 (4)	0.47745 (9)	0.3474 (3)	0.0576 (9)	
H28C	1.1441	0.4913	0.4064	0.069*	
H28D	1.1244	0.4635	0.3021	0.069*	
C29B	0.9715 (4)	0.50394 (10)	0.2689 (3)	0.0656 (10)	
H29C	0.9017	0.4902	0.2083	0.079*	
H29D	0.9177	0.5175	0.3135	0.079*	
C30B	1.0493 (5)	0.53075 (11)	0.2134 (4)	0.0859 (14)	
H30D	1.1258	0.5425	0.2726	0.129*	
H30E	0.9818	0.5492	0.1719	0.129*	
H30F	1.0905	0.5179	0.1598	0.129*	

### Atomic displacement parameters (Å^2^)

**Table:**

	*U*^11^	*U*^22^	*U*^33^	*U*^12^	*U*^13^	*U*^23^
N1A	0.0341 (12)	0.0421 (13)	0.0419 (13)	0.0052 (10)	0.0169 (10)	0.0024 (11)
C2A	0.0292 (13)	0.0469 (16)	0.0260 (13)	0.0084 (12)	0.0122 (10)	0.0083 (11)
C3A	0.0258 (13)	0.0474 (16)	0.0296 (13)	0.0075 (12)	0.0118 (10)	0.0063 (12)
N4A	0.0250 (11)	0.0503 (14)	0.0337 (12)	0.0038 (10)	0.0108 (9)	−0.0028 (10)
C5A	0.0223 (12)	0.0589 (18)	0.0376 (15)	0.0078 (12)	0.0107 (11)	−0.0016 (13)
N6A	0.0259 (12)	0.088 (2)	0.0518 (16)	0.0034 (13)	0.0112 (11)	−0.0151 (15)
N7A	0.0275 (13)	0.098 (2)	0.0566 (17)	−0.0048 (14)	0.0095 (12)	−0.0279 (16)
C8A	0.0278 (14)	0.070 (2)	0.0402 (16)	−0.0036 (14)	0.0089 (12)	−0.0121 (15)
C9A	0.0435 (17)	0.068 (2)	0.0461 (18)	−0.0057 (16)	0.0114 (14)	−0.0166 (16)
C10A	0.0460 (17)	0.0547 (19)	0.0404 (16)	0.0033 (14)	0.0168 (14)	−0.0081 (14)
C11A	0.0380 (15)	0.0485 (17)	0.0343 (14)	0.0060 (13)	0.0158 (12)	0.0021 (13)
C12A	0.0448 (17)	0.0503 (17)	0.0397 (16)	0.0137 (14)	0.0209 (13)	−0.0005 (14)
C13A	0.0325 (15)	0.0553 (18)	0.0421 (16)	0.0124 (13)	0.0190 (12)	0.0073 (14)
C14A	0.0269 (13)	0.0495 (16)	0.0338 (14)	0.0080 (12)	0.0128 (11)	0.0091 (12)
C15A	0.0276 (14)	0.0588 (19)	0.0433 (16)	0.0025 (13)	0.0173 (12)	0.0101 (14)
C16A	0.0358 (15)	0.0516 (18)	0.0467 (17)	−0.0044 (13)	0.0146 (13)	0.0066 (14)
C17A	0.0440 (17)	0.0439 (17)	0.0520 (18)	0.0036 (14)	0.0219 (14)	0.0020 (14)
C18A	0.0235 (12)	0.0498 (16)	0.0361 (14)	0.0059 (12)	0.0138 (11)	0.0008 (12)
C19A	0.0336 (15)	0.0588 (19)	0.0404 (16)	0.0165 (14)	0.0085 (12)	0.0025 (14)
C20A	0.0431 (17)	0.0523 (19)	0.0485 (18)	0.0200 (14)	0.0102 (14)	0.0013 (15)
C21A	0.0360 (15)	0.0443 (16)	0.0407 (16)	0.0049 (12)	0.0116 (12)	−0.0026 (13)
C22A	0.0299 (14)	0.0400 (15)	0.0398 (15)	0.0063 (12)	0.0062 (11)	0.0050 (12)
C23A	0.0281 (13)	0.0406 (15)	0.0445 (16)	0.0052 (11)	0.0159 (12)	0.0000 (13)
O24A	0.0531 (13)	0.0459 (12)	0.0474 (12)	0.0149 (10)	0.0050 (10)	−0.0077 (10)
C25A	0.0543 (18)	0.0430 (17)	0.0474 (17)	0.0090 (14)	0.0185 (14)	−0.0008 (14)
C26A	0.0490 (18)	0.0460 (18)	0.0493 (18)	0.0030 (14)	0.0129 (14)	−0.0024 (14)
C27A	0.070 (2)	0.0520 (19)	0.0453 (18)	0.0074 (17)	0.0156 (17)	−0.0043 (15)
C28A	0.070 (2)	0.056 (2)	0.062 (2)	−0.0069 (18)	0.0226 (19)	−0.0114 (17)
C29A	0.108 (4)	0.076 (3)	0.067 (3)	−0.002 (3)	0.009 (3)	−0.016 (2)
C30A	0.133 (5)	0.098 (4)	0.087 (3)	−0.013 (3)	0.023 (3)	−0.036 (3)
N1B	0.0279 (11)	0.0406 (13)	0.0321 (11)	0.0008 (9)	0.0120 (9)	−0.0025 (10)
C2B	0.0236 (12)	0.0303 (13)	0.0306 (13)	0.0004 (10)	0.0115 (10)	0.0026 (10)
C3B	0.0223 (12)	0.0351 (13)	0.0304 (13)	−0.0001 (10)	0.0105 (10)	0.0028 (11)
N4B	0.0196 (10)	0.0335 (11)	0.0320 (11)	0.0004 (8)	0.0100 (8)	−0.0002 (9)
C5B	0.0210 (12)	0.0381 (14)	0.0344 (13)	−0.0014 (11)	0.0092 (10)	0.0014 (11)
N6B	0.0230 (11)	0.0446 (13)	0.0475 (14)	0.0012 (10)	0.0134 (10)	−0.0035 (11)
N7B	0.0278 (11)	0.0421 (13)	0.0454 (14)	0.0047 (10)	0.0129 (10)	−0.0038 (11)
C8B	0.0287 (13)	0.0369 (14)	0.0306 (13)	0.0067 (11)	0.0115 (10)	−0.0003 (11)
C9B	0.0374 (14)	0.0340 (14)	0.0388 (15)	0.0041 (12)	0.0149 (12)	−0.0025 (12)
C10B	0.0392 (15)	0.0345 (14)	0.0369 (14)	−0.0034 (12)	0.0117 (12)	−0.0055 (12)
C11B	0.0312 (13)	0.0334 (14)	0.0337 (14)	−0.0021 (11)	0.0123 (11)	0.0000 (11)
C12B	0.0310 (14)	0.0456 (16)	0.0431 (16)	−0.0102 (12)	0.0122 (12)	−0.0054 (13)
C13B	0.0226 (13)	0.0521 (17)	0.0451 (16)	−0.0067 (12)	0.0125 (11)	−0.0030 (13)
C14B	0.0233 (12)	0.0404 (15)	0.0369 (14)	−0.0017 (11)	0.0114 (11)	0.0004 (12)
C15B	0.0271 (14)	0.0523 (18)	0.0492 (17)	0.0027 (12)	0.0186 (12)	0.0001 (14)
C16B	0.0399 (16)	0.0515 (18)	0.0476 (17)	0.0040 (14)	0.0222 (14)	−0.0098 (14)
C17B	0.0383 (15)	0.0428 (16)	0.0388 (15)	−0.0030 (13)	0.0160 (12)	−0.0065 (13)
C18B	0.0213 (12)	0.0371 (14)	0.0330 (13)	−0.0035 (10)	0.0119 (10)	0.0004 (11)
C19B	0.0280 (13)	0.0440 (16)	0.0391 (15)	−0.0037 (12)	0.0062 (11)	0.0018 (12)
C20B	0.0370 (15)	0.0488 (17)	0.0374 (15)	−0.0100 (13)	0.0015 (12)	−0.0055 (13)
C21B	0.0451 (16)	0.0339 (14)	0.0394 (15)	−0.0044 (12)	0.0156 (13)	−0.0037 (12)
C22B	0.0325 (14)	0.0369 (15)	0.0357 (14)	0.0039 (11)	0.0074 (11)	0.0022 (12)
C23B	0.0270 (13)	0.0426 (15)	0.0282 (13)	−0.0042 (11)	0.0078 (10)	−0.0036 (11)
O24B	0.0715 (15)	0.0361 (11)	0.0516 (13)	−0.0002 (11)	0.0098 (11)	−0.0075 (10)
C25B	0.069 (2)	0.0391 (17)	0.062 (2)	0.0068 (16)	0.0263 (18)	−0.0044 (15)
C26B	0.056 (2)	0.059 (2)	0.056 (2)	0.0071 (16)	0.0175 (16)	−0.0039 (16)
C27B	0.055 (2)	0.058 (2)	0.064 (2)	0.0063 (17)	0.0142 (17)	−0.0074 (18)
C28B	0.070 (2)	0.057 (2)	0.0460 (18)	0.0054 (18)	0.0161 (17)	−0.0057 (16)
C29B	0.073 (2)	0.063 (2)	0.058 (2)	0.0058 (19)	0.0131 (18)	−0.0108 (18)
C30B	0.120 (4)	0.073 (3)	0.070 (3)	0.012 (3)	0.037 (3)	0.006 (2)

### Geometric parameters (Å, º)

**Table:**

N1A—C17A	1.318 (4)	N1B—C17B	1.319 (3)
N1A—C2A	1.353 (3)	N1B—C2B	1.353 (3)
C2A—C14A	1.421 (3)	C2B—C14B	1.421 (3)
C2A—C3A	1.428 (4)	C2B—C3B	1.433 (3)
C3A—C11A	1.394 (4)	C3B—C11B	1.389 (3)
C3A—N4A	1.418 (3)	C3B—N4B	1.417 (3)
N4A—C5A	1.392 (3)	N4B—C5B	1.388 (3)
N4A—C8A	1.396 (3)	N4B—C8B	1.393 (3)
C5A—N6A	1.315 (3)	C5B—N6B	1.321 (3)
C5A—C18A	1.467 (4)	C5B—C18B	1.462 (4)
N6A—N7A	1.369 (4)	N6B—N7B	1.374 (3)
N7A—C8A	1.312 (4)	N7B—C8B	1.319 (3)
C8A—C9A	1.413 (4)	C8B—C9B	1.413 (4)
C9A—C10A	1.342 (4)	C9B—C10B	1.350 (4)
C9A—H9A	0.9500	C9B—H9B	0.9500
C10A—C11A	1.429 (4)	C10B—C11B	1.433 (4)
C10A—H10A	0.9500	C10B—H10B	0.9500
C11A—C12A	1.428 (4)	C11B—C12B	1.430 (3)
C12A—C13A	1.343 (4)	C12B—C13B	1.359 (4)
C12A—H12A	0.9500	C12B—H12B	0.9500
C13A—C14A	1.420 (4)	C13B—C14B	1.412 (4)
C13A—H13A	0.9500	C13B—H13B	0.9500
C14A—C15A	1.400 (4)	C14B—C15B	1.404 (4)
C15A—C16A	1.358 (4)	C15B—C16B	1.357 (4)
C15A—H15A	0.9500	C15B—H15B	0.9500
C16A—C17A	1.403 (4)	C16B—C17B	1.406 (4)
C16A—H16A	0.9500	C16B—H16B	0.9500
C17A—H17A	0.9500	C17B—H17B	0.9500
C18A—C19A	1.383 (4)	C18B—C23B	1.384 (3)
C18A—C23A	1.397 (4)	C18B—C19B	1.392 (4)
C19A—C20A	1.374 (4)	C19B—C20B	1.378 (4)
C19A—H19A	0.9500	C19B—H19B	0.9500
C20A—C21A	1.383 (4)	C20B—C21B	1.388 (4)
C20A—H20A	0.9500	C20B—H20B	0.9500
C21A—O24A	1.369 (3)	C21B—O24B	1.367 (3)
C21A—C22A	1.389 (4)	C21B—C22B	1.387 (4)
C22A—C23A	1.370 (4)	C22B—C23B	1.379 (4)
C22A—H22A	0.9500	C22B—H22B	0.9500
C23A—H23A	0.9500	C23B—H23B	0.9500
O24A—C25A	1.426 (3)	O24B—C25B	1.430 (4)
C25A—C26A	1.508 (4)	C25B—C26B	1.495 (5)
C25A—H25A	0.9900	C25B—H25C	0.9900
C25A—H25B	0.9900	C25B—H25D	0.9900
C26A—C27A	1.512 (4)	C26B—C27B	1.523 (5)
C26A—H26A	0.9900	C26B—H26C	0.9900
C26A—H26B	0.9900	C26B—H26D	0.9900
C27A—C28A	1.497 (4)	C27B—C28B	1.520 (5)
C27A—H27A	0.9900	C27B—H27C	0.9900
C27A—H27B	0.9900	C27B—H27D	0.9900
C28A—C29A	1.537 (5)	C28B—C29B	1.517 (5)
C28A—H28A	0.9900	C28B—H28C	0.9900
C28A—H28B	0.9900	C28B—H28D	0.9900
C29A—C30A	1.448 (5)	C29B—C30B	1.514 (5)
C29A—H29A	0.9900	C29B—H29C	0.9900
C29A—H29B	0.9900	C29B—H29D	0.9900
C30A—H30A	0.9800	C30B—H30D	0.9800
C30A—H30B	0.9800	C30B—H30E	0.9800
C30A—H30C	0.9800	C30B—H30F	0.9800
			
C17A—N1A—C2A	118.1 (2)	C17B—N1B—C2B	117.3 (2)
N1A—C2A—C14A	121.6 (3)	N1B—C2B—C14B	122.8 (2)
N1A—C2A—C3A	119.8 (2)	N1B—C2B—C3B	119.0 (2)
C14A—C2A—C3A	118.6 (2)	C14B—C2B—C3B	118.1 (2)
C11A—C3A—N4A	116.7 (2)	C11B—C3B—N4B	117.0 (2)
C11A—C3A—C2A	120.1 (2)	C11B—C3B—C2B	120.2 (2)
N4A—C3A—C2A	123.0 (2)	N4B—C3B—C2B	122.7 (2)
C5A—N4A—C8A	103.8 (2)	C5B—N4B—C8B	104.2 (2)
C5A—N4A—C3A	136.4 (2)	C5B—N4B—C3B	135.5 (2)
C8A—N4A—C3A	119.6 (2)	C8B—N4B—C3B	120.3 (2)
N6A—C5A—N4A	108.9 (3)	N6B—C5B—N4B	109.0 (2)
N6A—C5A—C18A	119.3 (2)	N6B—C5B—C18B	121.0 (2)
N4A—C5A—C18A	131.1 (2)	N4B—C5B—C18B	129.4 (2)
C5A—N6A—N7A	109.7 (2)	C5B—N6B—N7B	109.3 (2)
C8A—N7A—N6A	106.9 (2)	C8B—N7B—N6B	106.8 (2)
N7A—C8A—N4A	110.6 (3)	N7B—C8B—N4B	110.5 (2)
N7A—C8A—C9A	128.0 (3)	N7B—C8B—C9B	128.2 (2)
N4A—C8A—C9A	121.3 (3)	N4B—C8B—C9B	120.9 (2)
C10A—C9A—C8A	118.8 (3)	C10B—C9B—C8B	118.5 (2)
C10A—C9A—H9A	120.6	C10B—C9B—H9B	120.7
C8A—C9A—H9A	120.6	C8B—C9B—H9B	120.7
C9A—C10A—C11A	120.6 (3)	C9B—C10B—C11B	121.0 (3)
C9A—C10A—H10A	119.7	C9B—C10B—H10B	119.5
C11A—C10A—H10A	119.7	C11B—C10B—H10B	119.5
C3A—C11A—C12A	118.9 (3)	C3B—C11B—C12B	119.0 (2)
C3A—C11A—C10A	121.6 (3)	C3B—C11B—C10B	120.9 (2)
C12A—C11A—C10A	119.4 (3)	C12B—C11B—C10B	120.0 (2)
C13A—C12A—C11A	121.9 (3)	C13B—C12B—C11B	121.1 (3)
C13A—C12A—H12A	119.1	C13B—C12B—H12B	119.5
C11A—C12A—H12A	119.1	C11B—C12B—H12B	119.5
C12A—C13A—C14A	120.3 (2)	C12B—C13B—C14B	120.5 (2)
C12A—C13A—H13A	119.9	C12B—C13B—H13B	119.8
C14A—C13A—H13A	119.9	C14B—C13B—H13B	119.8
C15A—C14A—C13A	122.3 (2)	C15B—C14B—C13B	122.8 (2)
C15A—C14A—C2A	117.9 (3)	C15B—C14B—C2B	117.1 (2)
C13A—C14A—C2A	119.8 (3)	C13B—C14B—C2B	120.0 (2)
C16A—C15A—C14A	119.9 (3)	C16B—C15B—C14B	119.6 (2)
C16A—C15A—H15A	120.1	C16B—C15B—H15B	120.2
C14A—C15A—H15A	120.1	C14B—C15B—H15B	120.2
C15A—C16A—C17A	118.2 (3)	C15B—C16B—C17B	119.0 (3)
C15A—C16A—H16A	120.9	C15B—C16B—H16B	120.5
C17A—C16A—H16A	120.9	C17B—C16B—H16B	120.5
N1A—C17A—C16A	124.1 (3)	N1B—C17B—C16B	123.8 (3)
N1A—C17A—H17A	117.9	N1B—C17B—H17B	118.1
C16A—C17A—H17A	117.9	C16B—C17B—H17B	118.1
C19A—C18A—C23A	118.0 (3)	C23B—C18B—C19B	118.8 (2)
C19A—C18A—C5A	119.2 (2)	C23B—C18B—C5B	121.3 (2)
C23A—C18A—C5A	122.4 (3)	C19B—C18B—C5B	119.7 (2)
C20A—C19A—C18A	121.4 (3)	C20B—C19B—C18B	120.1 (3)
C20A—C19A—H19A	119.3	C20B—C19B—H19B	119.9
C18A—C19A—H19A	119.3	C18B—C19B—H19B	119.9
C19A—C20A—C21A	120.2 (3)	C19B—C20B—C21B	120.4 (3)
C19A—C20A—H20A	119.9	C19B—C20B—H20B	119.8
C21A—C20A—H20A	119.9	C21B—C20B—H20B	119.8
O24A—C21A—C20A	125.3 (3)	O24B—C21B—C22B	124.9 (3)
O24A—C21A—C22A	115.7 (2)	O24B—C21B—C20B	115.2 (2)
C20A—C21A—C22A	119.0 (3)	C22B—C21B—C20B	119.8 (3)
C23A—C22A—C21A	120.5 (3)	C23B—C22B—C21B	119.3 (2)
C23A—C22A—H22A	119.7	C23B—C22B—H22B	120.4
C21A—C22A—H22A	119.7	C21B—C22B—H22B	120.4
C22A—C23A—C18A	120.8 (3)	C22B—C23B—C18B	121.5 (2)
C22A—C23A—H23A	119.6	C22B—C23B—H23B	119.3
C18A—C23A—H23A	119.6	C18B—C23B—H23B	119.3
C21A—O24A—C25A	118.0 (2)	C21B—O24B—C25B	119.1 (2)
O24A—C25A—C26A	107.6 (2)	O24B—C25B—C26B	111.9 (3)
O24A—C25A—H25A	110.2	O24B—C25B—H25C	109.2
C26A—C25A—H25A	110.2	C26B—C25B—H25C	109.2
O24A—C25A—H25B	110.2	O24B—C25B—H25D	109.2
C26A—C25A—H25B	110.2	C26B—C25B—H25D	109.2
H25A—C25A—H25B	108.5	H25C—C25B—H25D	107.9
C25A—C26A—C27A	111.6 (3)	C25B—C26B—C27B	112.2 (3)
C25A—C26A—H26A	109.3	C25B—C26B—H26C	109.2
C27A—C26A—H26A	109.3	C27B—C26B—H26C	109.2
C25A—C26A—H26B	109.3	C25B—C26B—H26D	109.2
C27A—C26A—H26B	109.3	C27B—C26B—H26D	109.2
H26A—C26A—H26B	108.0	H26C—C26B—H26D	107.9
C28A—C27A—C26A	115.1 (3)	C28B—C27B—C26B	114.1 (3)
C28A—C27A—H27A	108.5	C28B—C27B—H27C	108.7
C26A—C27A—H27A	108.5	C26B—C27B—H27C	108.7
C28A—C27A—H27B	108.5	C28B—C27B—H27D	108.7
C26A—C27A—H27B	108.5	C26B—C27B—H27D	108.7
H27A—C27A—H27B	107.5	H27C—C27B—H27D	107.6
C27A—C28A—C29A	113.5 (3)	C29B—C28B—C27B	113.3 (3)
C27A—C28A—H28A	108.9	C29B—C28B—H28C	108.9
C29A—C28A—H28A	108.9	C27B—C28B—H28C	108.9
C27A—C28A—H28B	108.9	C29B—C28B—H28D	108.9
C29A—C28A—H28B	108.9	C27B—C28B—H28D	108.9
H28A—C28A—H28B	107.7	H28C—C28B—H28D	107.7
C30A—C29A—C28A	114.4 (4)	C30B—C29B—C28B	112.5 (3)
C30A—C29A—H29A	108.7	C30B—C29B—H29C	109.1
C28A—C29A—H29A	108.7	C28B—C29B—H29C	109.1
C30A—C29A—H29B	108.7	C30B—C29B—H29D	109.1
C28A—C29A—H29B	108.7	C28B—C29B—H29D	109.1
H29A—C29A—H29B	107.6	H29C—C29B—H29D	107.8
C29A—C30A—H30A	109.5	C29B—C30B—H30D	109.5
C29A—C30A—H30B	109.5	C29B—C30B—H30E	109.5
H30A—C30A—H30B	109.5	H30D—C30B—H30E	109.5
C29A—C30A—H30C	109.5	C29B—C30B—H30F	109.5
H30A—C30A—H30C	109.5	H30D—C30B—H30F	109.5
H30B—C30A—H30C	109.5	H30E—C30B—H30F	109.5
			
C17A—N1A—C2A—C14A	5.1 (4)	C17B—N1B—C2B—C14B	−5.5 (4)
C17A—N1A—C2A—C3A	−178.1 (2)	C17B—N1B—C2B—C3B	178.4 (2)
N1A—C2A—C3A—C11A	−169.2 (2)	N1B—C2B—C3B—C11B	163.5 (2)
C14A—C2A—C3A—C11A	7.7 (4)	C14B—C2B—C3B—C11B	−12.8 (4)
N1A—C2A—C3A—N4A	5.0 (4)	N1B—C2B—C3B—N4B	−12.3 (4)
C14A—C2A—C3A—N4A	−178.1 (2)	C14B—C2B—C3B—N4B	171.5 (2)
C11A—C3A—N4A—C5A	−171.1 (3)	C11B—C3B—N4B—C5B	167.9 (3)
C2A—C3A—N4A—C5A	14.5 (5)	C2B—C3B—N4B—C5B	−16.2 (4)
C11A—C3A—N4A—C8A	14.2 (4)	C11B—C3B—N4B—C8B	−13.9 (3)
C2A—C3A—N4A—C8A	−160.2 (3)	C2B—C3B—N4B—C8B	162.0 (2)
C8A—N4A—C5A—N6A	3.3 (3)	C8B—N4B—C5B—N6B	−3.0 (3)
C3A—N4A—C5A—N6A	−171.9 (3)	C3B—N4B—C5B—N6B	175.4 (3)
C8A—N4A—C5A—C18A	−166.3 (3)	C8B—N4B—C5B—C18B	168.3 (3)
C3A—N4A—C5A—C18A	18.4 (6)	C3B—N4B—C5B—C18B	−13.3 (5)
N4A—C5A—N6A—N7A	−1.7 (4)	N4B—C5B—N6B—N7B	1.8 (3)
C18A—C5A—N6A—N7A	169.4 (3)	C18B—C5B—N6B—N7B	−170.3 (2)
C5A—N6A—N7A—C8A	−0.9 (4)	C5B—N6B—N7B—C8B	0.2 (3)
N6A—N7A—C8A—N4A	3.1 (4)	N6B—N7B—C8B—N4B	−2.2 (3)
N6A—N7A—C8A—C9A	−173.1 (3)	N6B—N7B—C8B—C9B	171.2 (3)
C5A—N4A—C8A—N7A	−3.9 (4)	C5B—N4B—C8B—N7B	3.2 (3)
C3A—N4A—C8A—N7A	172.3 (3)	C3B—N4B—C8B—N7B	−175.5 (2)
C5A—N4A—C8A—C9A	172.5 (3)	C5B—N4B—C8B—C9B	−170.7 (2)
C3A—N4A—C8A—C9A	−11.3 (4)	C3B—N4B—C8B—C9B	10.6 (4)
N7A—C8A—C9A—C10A	176.7 (4)	N7B—C8B—C9B—C10B	−173.7 (3)
N4A—C8A—C9A—C10A	1.0 (5)	N4B—C8B—C9B—C10B	−0.9 (4)
C8A—C9A—C10A—C11A	5.7 (5)	C8B—C9B—C10B—C11B	−4.9 (4)
N4A—C3A—C11A—C12A	177.3 (2)	N4B—C3B—C11B—C12B	−173.6 (2)
C2A—C3A—C11A—C12A	−8.1 (4)	C2B—C3B—C11B—C12B	10.4 (4)
N4A—C3A—C11A—C10A	−7.8 (4)	N4B—C3B—C11B—C10B	8.2 (4)
C2A—C3A—C11A—C10A	166.8 (3)	C2B—C3B—C11B—C10B	−167.8 (2)
C9A—C10A—C11A—C3A	−2.2 (5)	C9B—C10B—C11B—C3B	1.1 (4)
C9A—C10A—C11A—C12A	172.6 (3)	C9B—C10B—C11B—C12B	−177.1 (3)
C3A—C11A—C12A—C13A	3.5 (4)	C3B—C11B—C12B—C13B	−1.4 (4)
C10A—C11A—C12A—C13A	−171.5 (3)	C10B—C11B—C12B—C13B	176.8 (3)
C11A—C12A—C13A—C14A	1.5 (4)	C11B—C12B—C13B—C14B	−5.0 (4)
C12A—C13A—C14A—C15A	177.5 (3)	C12B—C13B—C14B—C15B	−173.9 (3)
C12A—C13A—C14A—C2A	−1.9 (4)	C12B—C13B—C14B—C2B	2.3 (4)
N1A—C2A—C14A—C15A	−5.2 (4)	N1B—C2B—C14B—C15B	6.8 (4)
C3A—C2A—C14A—C15A	177.9 (2)	C3B—C2B—C14B—C15B	−177.1 (2)
N1A—C2A—C14A—C13A	174.2 (2)	N1B—C2B—C14B—C13B	−169.7 (2)
C3A—C2A—C14A—C13A	−2.7 (4)	C3B—C2B—C14B—C13B	6.4 (4)
C13A—C14A—C15A—C16A	−177.7 (3)	C13B—C14B—C15B—C16B	173.8 (3)
C2A—C14A—C15A—C16A	1.7 (4)	C2B—C14B—C15B—C16B	−2.6 (4)
C14A—C15A—C16A—C17A	1.7 (4)	C14B—C15B—C16B—C17B	−2.3 (4)
C2A—N1A—C17A—C16A	−1.5 (4)	C2B—N1B—C17B—C16B	0.1 (4)
C15A—C16A—C17A—N1A	−1.9 (5)	C15B—C16B—C17B—N1B	3.8 (5)
N6A—C5A—C18A—C19A	45.1 (4)	N6B—C5B—C18B—C23B	120.5 (3)
N4A—C5A—C18A—C19A	−146.1 (3)	N4B—C5B—C18B—C23B	−49.9 (4)
N6A—C5A—C18A—C23A	−127.9 (3)	N6B—C5B—C18B—C19B	−54.7 (4)
N4A—C5A—C18A—C23A	40.8 (5)	N4B—C5B—C18B—C19B	135.0 (3)
C23A—C18A—C19A—C20A	−1.6 (4)	C23B—C18B—C19B—C20B	1.9 (4)
C5A—C18A—C19A—C20A	−175.0 (3)	C5B—C18B—C19B—C20B	177.1 (2)
C18A—C19A—C20A—C21A	0.4 (5)	C18B—C19B—C20B—C21B	−0.5 (4)
C19A—C20A—C21A—O24A	−179.0 (3)	C19B—C20B—C21B—O24B	177.5 (3)
C19A—C20A—C21A—C22A	0.8 (5)	C19B—C20B—C21B—C22B	−1.2 (4)
O24A—C21A—C22A—C23A	179.1 (2)	O24B—C21B—C22B—C23B	−177.0 (3)
C20A—C21A—C22A—C23A	−0.8 (4)	C20B—C21B—C22B—C23B	1.6 (4)
C21A—C22A—C23A—C18A	−0.5 (4)	C21B—C22B—C23B—C18B	−0.2 (4)
C19A—C18A—C23A—C22A	1.7 (4)	C19B—C18B—C23B—C22B	−1.5 (4)
C5A—C18A—C23A—C22A	174.8 (2)	C5B—C18B—C23B—C22B	−176.6 (2)
C20A—C21A—O24A—C25A	−1.8 (4)	C22B—C21B—O24B—C25B	−9.2 (4)
C22A—C21A—O24A—C25A	178.3 (3)	C20B—C21B—O24B—C25B	172.1 (3)
C21A—O24A—C25A—C26A	175.9 (2)	C21B—O24B—C25B—C26B	−88.3 (3)
O24A—C25A—C26A—C27A	179.0 (3)	O24B—C25B—C26B—C27B	−178.3 (3)
C25A—C26A—C27A—C28A	178.0 (3)	C25B—C26B—C27B—C28B	164.8 (3)
C26A—C27A—C28A—C29A	178.9 (3)	C26B—C27B—C28B—C29B	177.5 (3)
C27A—C28A—C29A—C30A	176.8 (4)	C27B—C28B—C29B—C30B	178.4 (3)

## References

[bb1] Altomare, A., Burla, M. C., Camalli, M., Cascarano, G. L., Giacovazzo, C., Guagliardi, A., Moliterni, A. G. G., Polidori, G. & Spagna, R. (1999). *J. Appl. Cryst.* **32**, 115–119.

[bb2] Caronna, T., Castiglione, F., Famulari, A., Fontana, F., Malpezzi, L., Mele, A., Mendola, D. & Sora, I. N. (2012). *Molecules*, **17**, 463–479. 10.3390/molecules17010463 PMC6268832 22222906

[bb3] Cristiano, R., Gallardo, H., Bortoluzzi, A. J., Bechtold, I. H., Campos, C. E. M. & Longo, R. L. (2008). *Chem. Commun.* pp. 5134. 10.1039/b810680k 18956046

[bb4] Dräger, M. & Gattow, G. (1971). *Acta Chem. Scand.* **25**, 761–762.

[bb5] Enraf–Nonius (1989). *CAD-4 Software*. Enraf–Nonius, Delft, The Netherlands.

[bb6] Glang, S., Rieth, T., Borchmann, D., Fortunati, I., Signorini, R. & Detert, H. (2014). *Eur. J. Org. Chem.* pp. 3116–3126.

[bb7] Herget, K., Schollmeyer, D. & Detert, H. (2013). *Acta Cryst.* E**69**, o365–o366. 10.1107/S1600536813003498 PMC3588459 23476555

[bb8] Huisgen, R., Sauer, J. & Seidel, M. (1960). *Chem. Ber.* **93**, 2885–2891.

[bb9] Lewis, J. & O’Donoghue, T. D. (1980). *J. Chem. Soc. Dalton Trans.* pp. 736–742.

[bb10] Preis, J., Schollmeyer, D. & Detert, H. (2011*a*). *Acta Cryst.* E**67**, o987. 10.1107/S1600536811010683 PMC3099827 21754244

[bb11] Preis, J., Schollmeyer, D. & Detert, H. (2011*b*). *Acta Cryst.* E**67**, o2551. 10.1107/S1600536811035288 PMC3201469 22058729

[bb12] Rieth, T., Marszalek, T., Pisula, W. & Detert, H. (2014). *Chem. Eur. J.* **20**, 5000–5006. 10.1002/chem.201400034 24623447

[bb13] Schollmeyer, D. & Detert, H. (2014). *Acta Cryst.* E**70**, o247. 10.1107/S1600536814002153 PMC3998472 24764967

[bb14] Sheldrick, G. M. (2015). *Acta Cryst.* C**71**, 3–8.

[bb15] Spek, A. L. (2009). *Acta Cryst.* D**65**, 148–155. 10.1107/S090744490804362X PMC2631630 19171970

[bb16] Upadhyay, G. M., Talele, H. R., Sahoo, S. & Bedekar, A. V. (2014). *Tetrahedron Lett.* **55**, 5394–5399.

