# Supplementary figures and images for: Crystal structure of 11-[4-(hex­yloxy)phen­yl]-1,2,4-triazolo[4,3-a][1,10]phenanthroline
Source: Acta Crystallogr E Crystallogr Commun. 2015 Jun 27;71(Pt 7):o521–2. doi: 10.1107/S2056989015012025 (PMC4518990; doi:10.1107/S2056989015012025)

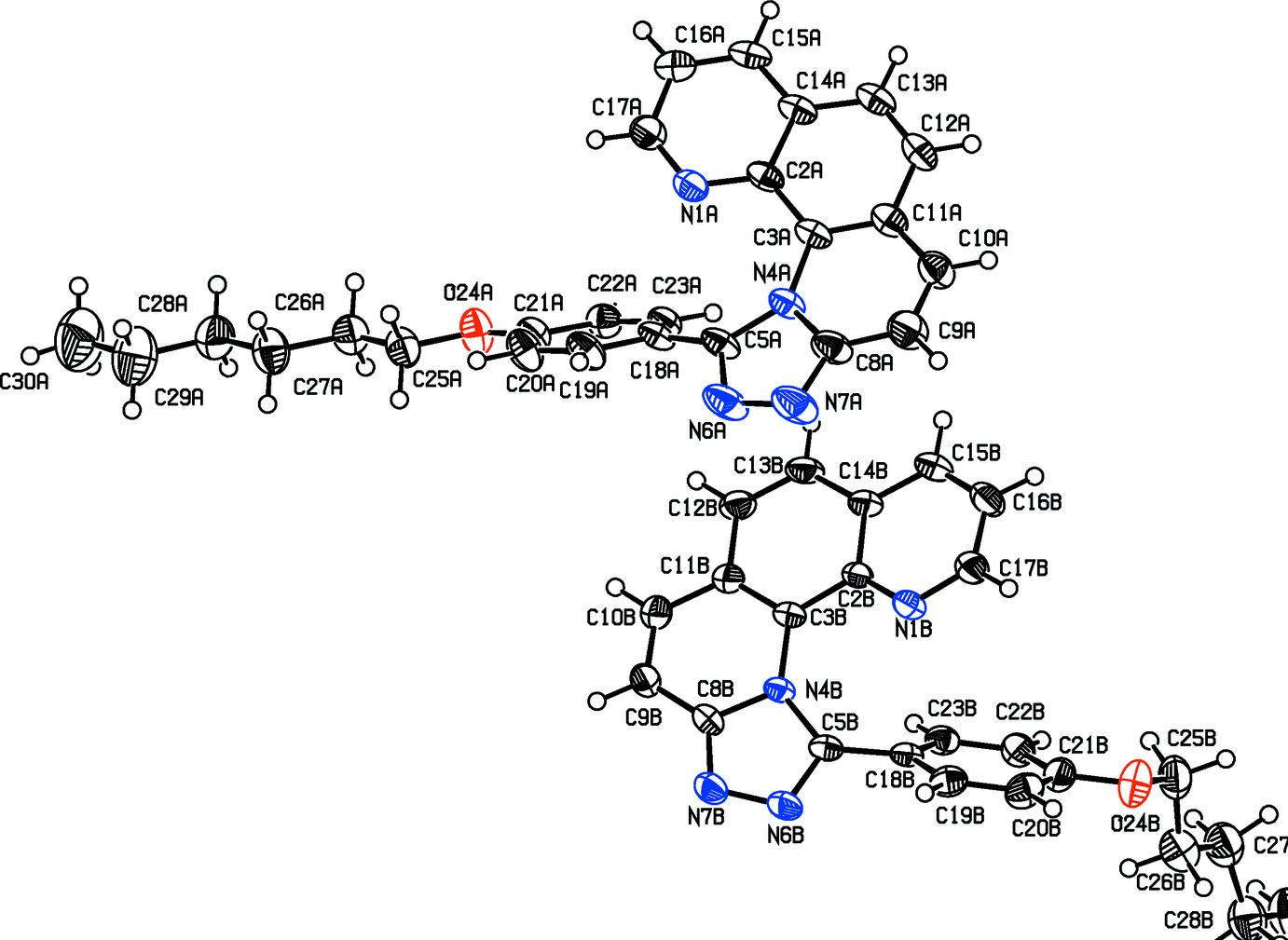

Supplement: Supplementary file 4 [file e-71-0o521-fig1.tif]

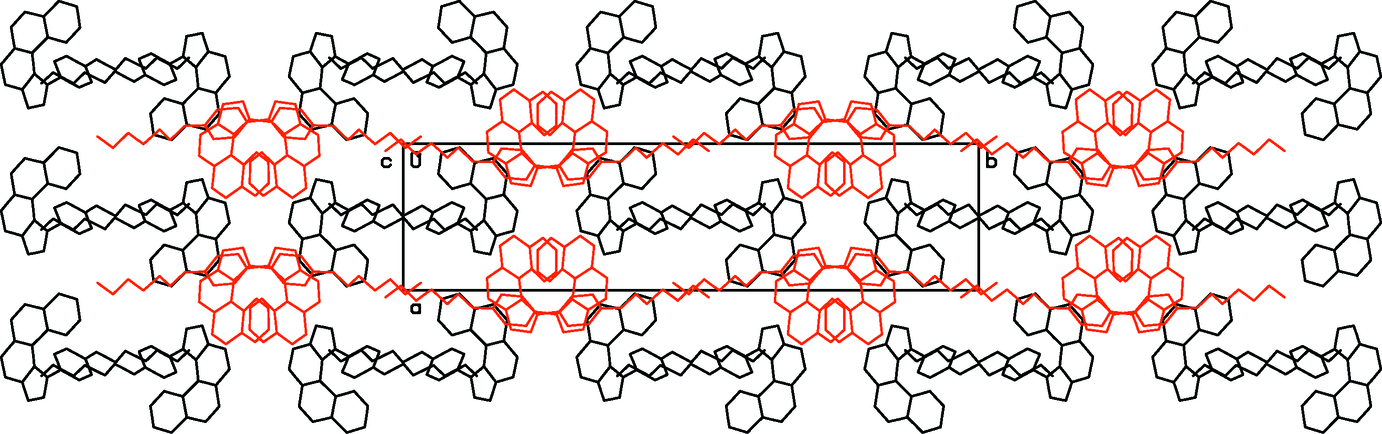

Supplement: Supplementary file 5 [file e-71-0o521-fig2.tif]
